# Recanalization of chronic long-segment occlusion of the internal carotid artery with endovascular and hybrid surgery

**DOI:** 10.1038/s41598-023-44406-x

**Published:** 2023-10-09

**Authors:** Wei Ren, Jiangyu Xue, Tongyuan Zhao, Gangqin Xu, Bowen Yang, Tianxiao Li, Bulang Gao

**Affiliations:** Stroke Center, Henan Provincial People’s Hospital, Zhengzhou University, 7 Weiwu Road, Zhengzhou, 450000 Henan China

**Keywords:** Diseases, Health care, Medical research, Neurology

## Abstract

To investigate the effect of endovascular and/or hybrid surgical recanalization on chronic long-segment occlusion of the internal carotid artery (ICA) and the effect of occlusion location on the recanalization rate and prognosis, 87 patients with chronic ICA occlusion treated with endovascular approach only or hybrid surgery were retrospectively enrolled. The duration of ICA occlusion ranged from 21 to 360 days (median 30). Type I occlusion (from the neck to below the cavernous segment) consisted of 46 (52.8%) patients while type II (from the neck to above the clinoid segment) of 41 (47.1%). Hybrid surgery was performed in 44 (50.6%) patients while endovascular recanalization only was conducted in the other 43 (49.4%). In all patients, the success rate of recanalization was 93.0% (40/43) for the endovascular approach and 95.5% for the hybrid surgical approach. In patients with type I occlusion, endovascular recanalization only was performed in 22 (47.8%) patients and hybrid surgery in 24 (52.2%), resulting in successful recanalization in all patients (100%). In patients with type II occlusion, the success rate of recanalization was 85.7% (18/21) for the endovascular approach only but 90% (18/20) for the hybrid surgery. The total success rate of recanalization was 94.3% (82/87) for all patients, 100% for type I occlusion, and 87.8% for type II occlusion. No significant (*P* = 0.12) differences existed in the recanalization rate between groups I and II. Clinical follow-up was performed in 82 (94.3%) patients 6–39 months (mean 16) after the surgery. Re-occlusion occurred in 0 in group I but in four (9.8%) in group II. The mRS was good with 0–2 in 38 (82.6%) patients in group I and in 27 (75%) patients in group II, with no significant (*P* = 0.78) difference. In conclusion, chronic long-segment ICA occlusion can be safely and efficiently recanalized with the endovascular and hybrid surgery. The location of ICA occlusion may have a critical role in determining the recanalization rate, and careful evaluation of the occlusion location may be helpful in increasing the prognosis of recanalization.

## Introduction

As one of the most important reasons for ischemic cerebral stroke^1–6^, chronic atherosclerotic occlusion of the internal carotid artery (ICA) has a grave natural history and is associated with a yearly risk of 6%-20% for ipsilateral recurrent stroke in spite of optimal medical treatment^7^. The annual recurrent stroke risk was higher in patients with known hemodynamic failure^8^ or impaired cerebrovascular reserve^9^. In a study evaluating the natural history of 167 patients with chronic ICA occlusion in a 5-year period^10^, neurological events occurred in 44 patients (26%), strokes took place in 30 (18%) patients, the cumulative 5-year stroke-free rate was 76%, and the overall 5-year cumulative survival rate was 63%. Patients with chronic ICA occlusion who originally presented with a stroke had a significantly unfavorable 4-year stroke-free rate (67%) compared with those who initially had transient ischemic attack (92%) or those who initially presented with no symptoms (89%)^10^. Intracranial-extracranial bypass surgery may not be able to reduce the ipsilateral recurrent stroke rate two years after the surgery as demonstrated in a prospective randomized controlled clinical trial^11^, which revealed a 2-year rate for the primary end point of all stroke and death as 21.0% for the surgical group and 22.7% for the nonsurgical group (best medical therapy), a 30-day rate for ipsilateral ischemic stroke as 14.4% (14/97) in the surgical group and 2.0% (2/98) in the nonsurgical group. Recanalization of non-acutely occluded ICA has been performed recently using the endovascular stent angioplasty or hybrid surgery, resulting in good effects and improved functional outcomes in patients with ICA occlusion compared to systemic thrombolysis or medical therapy alone^1–6,12–16^. The hybrid approach consists of endovascular stent angioplasty and carotid endarterectomy. In the study investigating the effect of hybrid and endovascular treatment of patients with chronic ICA occlusion^2^, the successful recanalization rate was 84.1% in patients with the hybrid surgery versus 51.4% in those with endovascular treatment. During the follow-up over 24 months, one patient died of a cardiovascular event, recurrent stroke took place in one unsuccessful patient, one patient had restenosis and one patient had reocclusion in the hybrid surgery group. In the endovascular group, three patients with unsuccessful recanalization experienced a recurrent transient ischemic stroke, one died, three patients had restenosis, and one patient had reocclusion. After investigating the long-term outcomes after endovascular recanalization in patients with chronic ICA occlusion^7^, it was found that the technical success and periprocedural complication rates of endovascular recanalization were acceptable and that the cumulative event rates of any stroke or death up to 7 years were more favorable in patients after successful recanalization compared to those without endovascular recanalization. Some factors may affect the safety and efficiency of the recanalization surgery for chronic ICA occlusion, especially the location of occlusion^17^. It was hypothesized that the location of chronic long-segment ICA occlusion caused by atherosclerosis might significantly affect the safety and efficiency of recanalization surgery for ICA occlusion. This study was consequently conducted to analyze the safety and efficiency of recanalization of long-segment atherosclerotic ICA occlusion and the effect of different occlusion locations on the recanalization rate and prognosis using the endovascular stent angioplasty and/or hybrid surgery.

## Materials and methods

### Subjects

This retrospective one-center study was approved by the ethics committee of Henan Provincial People’s Hospital, and informed consent has been waived by the same ethics committee because of the retrospective study design. All methods had been conducted in accordance to the relevant guidelines and regulations. Between January 2014 and December 2022, patients with chronic atherosclerotic occlusion of the ICA treated with hybrid surgery or endovascular treatment were retrospectively enrolled. The inclusion criteria were patients with chronic atherosclerotic occlusion of ICA confirmed by cerebral angiography, ICA occlusion duration ≥ 3 weeks, relevant ischemic symptoms related to the area supplied by the occluded ICA and refractory to medication alone, cerebral hemodynamic insufficiency or phase II hemodynamic failure with the mean transit time (MTT) > 4 s and decreased cerebral blood flow (CBF) (symptomatic side/asymptomatic side < 0.95) before recanalization on computed tomography perfusion (CTP), perfusion weighted imaging (PWI) of magnetic resonance imaging, or positron emission tomography (PET)^11,18^, good collateral compensation to or complete display of arteries distal to the location of ICA occlusion through collateral circulation, and treated with hybrid surgery or endovascular recanalization. Long-segment occlusion of ICA is usually caused by an atherosclerotic plaque and some thrombi formed proximal or distal to the plaque, and the thrombi may extend upwards or downwards to form a long segment of ICA occlusion. Hybrid surgery consisted of carotid endarterectomy and endovascular thrombectomy. Carotid endarterectomy was mainly performed to open the carotid artery and remove an atherosclerotic plaque located at the cervical carotid artery, and then, endovascular thrombectomy was conducted to remove thrombi formed distal to the plaque and extending upwards into the intracranial ICA with endovascular devices, thus recanalizing the occluded ICA. For endovascular surgery or thrombectomy, it was primarily conducted to remove an intracranial atherosclerotic plaque together with thrombi formed proximal to the plaque. The exclusion criteria were patients with acute ICA occlusion, irradiation history of the ipsilateral neck, intracranial hemorrhagic diseases within three months, and combined with severe cardiac or renal diseases. According to the division of ICA by Bouthillier et al.^19^, patients with ICA occlusion were divided into two types: type I (group I) with ICA occlusion from the neck segment to the cavernous segment while type II occlusion (group II) from the neck segment to the clinoid segment or beyond.

### Surgical procedures

Before the surgery, all patients were administered with dual antiplatelet therapy (aspirin 100 mg/d and clopidogrel 75 mg/d) for three days. The surgery included hybrid surgery and endovascular recanalization. The procedure was performed under general anesthesia. After puncture of the right femoral artery, a 6F or 8F arterial sheath was inserted into the femoral artery before a guiding catheter was navigated to near the bifurcation of the common carotid artery for cerebral angiography to confirm the occlusion of the ICA.

For hybrid surgery, the guiding catheter tip remained below the bifurcation of the common carotid artery before withdrawal of the guide wire. A cut was performed along the anterior margin of the sternocleidomastoid muscle before clamping the superior thyroid, common, internal and external carotid arteries above the tip of the guiding catheter. A longitudinal arteriotomy was conducted on the common and internal carotid arteries to remove the atherosclerotic plaque, and a microcatheter was navigated via the arteriotomy with the guidance of a guide wire whose tip was sent to the M3 segment of the ipsilateral middle cerebral artery. An exchange wire was sent into the artery before a 3F Fogarty balloon catheter was navigated to the middle cerebral artery for inflation to remove the long-segment thrombus in the ICA. If unobstructed blood flow was not established after thrombectomy in the occluded ICA proximal segment, a guide wire was navigated into the real vascular lumen under direct vision for guiding into a balloon catheter to expand the ICA before deployment of a stent to support the ICA. After restoration of the ICA blood flow, the arteriotomy was closed with 6–0 nylon sutures.

For endovascular recanalization, after the guiding catheter was navigated to the bifurcation of the common carotid artery, a guide wire was carefully sent through the occluded segment of ICA before an Echelon-10 micro-catheter was navigated to the distal segment of the ipsilateral middle cerebral artery under road mapping. A balloon catheter was navigated to expand the occluded ICA from distal to the proximal segments before a stent was deployed to support the ICA.

After both the hybrid and endovascular surgery, cerebral angiography and computed tomography were performed to confirm the patency of the ICA and possible intracranial hemorrhage. Dual antiplatelet therapy was performed with aspirin 100 mg/d and clopidogrel 75 mg/d as well as atorvastatin 20 mg/d for 6–12 months, and long-term application of aspirin 100 mg/d and atorvastin 20 mg/d was administered.

### Follow-up

Follow-up was performed at the clinics or through telephone 3, 6, 12 and 24 months after surgery, and re-occlusion of stented ICA segment on medical imaging or re-stroke events was recorded and evaluated with the modified Rankin scale (mRS): 0–2 for good prognosis and 3–5 for poor prognosis. Imaging follow-up with magnetic resonance imaging angiography or cerebral digital subtraction angiography was conducted once 6 months after the surgery, and once 1–3 years or at the time with recurrence of relevant ischemic symptoms related to the area supplied by the occluded ICA. Symptom improvement was defined as disappearance or decreased frequency of the occurrence of the symptoms related to the occluded ICA.

### Statistical analysis

The statistical analysis was performed with the SPSS version 22.0 (IBM, Chicago, IL, USA). Measurement data were presented as mean ± standard deviation and tested with the t-test if in normal distribution or as median and interquartile range and tested with the Mann Whitney U test. Categorical variables were expressed as frequency and percentage and tested with the Chi square test. The significant two-tailed *P* was set at *P* < 0.05.

## Results

Eighty-seven patients with chronic ICA occlusion were retrospectively enrolled, with an age range 35–72 years (mean 56.3 ± 7.8) and an M/F ratio of 63/24 (Table 1). The occlusion was located in the left ICA in 47 (54.0%) patients and in the right ICA in the other 40 (46.0%). The duration of ICA occlusion ranged from 21 to 360 days (median 30 and interquartile range 25–42 days). The transient ischemic attack symptoms were reversible, including slight limb weakness in 71 (81.6%) patients, unclear speech in 32 (36.8%), moderate headache in 7 (8.0%) and slight visual impairment in 8 (9.2%). The mRS score ranged 1–5 (median 3 and interquartile range 2–4). Type I consisted of 46 (52.8%) patients while type II of 41 (47.1%). No significant (*P* > 0.05) differences existed in the basic data between the two groups.

**Table 1:** Demographic data of patients in two groups.

Variables	Total (87)	Type I occlusion (46)	Type II occlusion (41)	*P*
Age (y)	56.3 ± 7.8	55.3 ± 9.1	57.8 ± 8.7	0.47
M/F	63/24	33/13	30/11	0.63
Smoking (n)	56 (64.4%)	29 (63.0%)	27 (65.9%)	0.42
Hypertension (n)	48 (55.2%)	26 (56.5%)	22 (53.7%)	0.37
Diabetes mellitus (n)	25 (28.7%)	14 (30.4%)	11 (26.8%)	0.52
Coronary heart disease (n)	17 (19.5%)	12 (26.1%)	5 (12.2%)	0.32
Atrial fibrillation (n)	4 (4.6%)	3 (6.5%)	1 (2.4%)	0.13
Past stroke (n)	15 (17.2%)	9 (19.6%)	6 (14.6%)	0.57
Duration of occlusion (d)	30 (21–360)	30 (21–360)	33 (24–360)	0.87
Left ICA occlusion	47 (54.0%)	28 (60.9%)	19 (46.3%)	0.32
Right ICA occlusion	40 (46.0%)	18 (39.1%)	22 (53.7%)	0.57
mRS	3 (1–5)	3 (1–5)	3 (1–5)	0.39
Symptoms				
Limb weakness (n)	71 (81.6%)	39 (84.8%)	32 (78.0%)	0.54
Unclear speech (n)	32 (36.8%)	12 (26.1%)	20 (48.8%)	0.16
Headache (n)	7 (8.0%)	3 (6.5%)	4 (9.8%)	0.33
Visual impairment (n)	8 (9.2%)	5 (10.9%)	3 (7.3%)	0.46

ICA, internal carotid artery; mRS, modified Rankin scale score.

**Figure 1:**
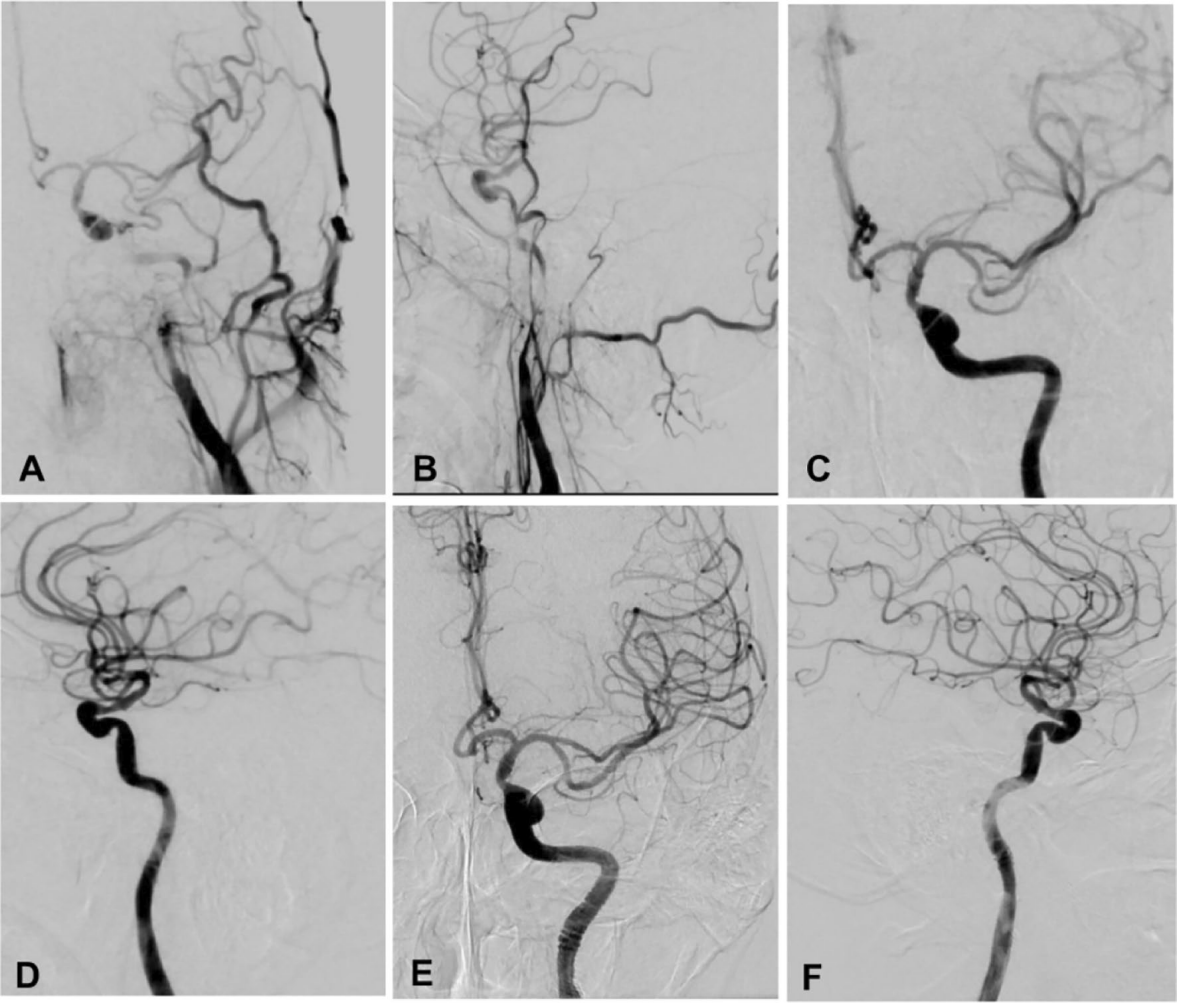
Endovascular recanalization was performed for a long atherosclerotic occlusion of the internal carotid artery (ICA). (**A** and **B**) The anterior–posterior (**A**) and lateral cerebral angiography showed long-segment occlusion of ICA. (**C** and **D**).Immediately after endovascular recanalization, the ICA was patent. (**E** and **F**) One-year follow-up revealed patent ICA.

**Figure 2:**
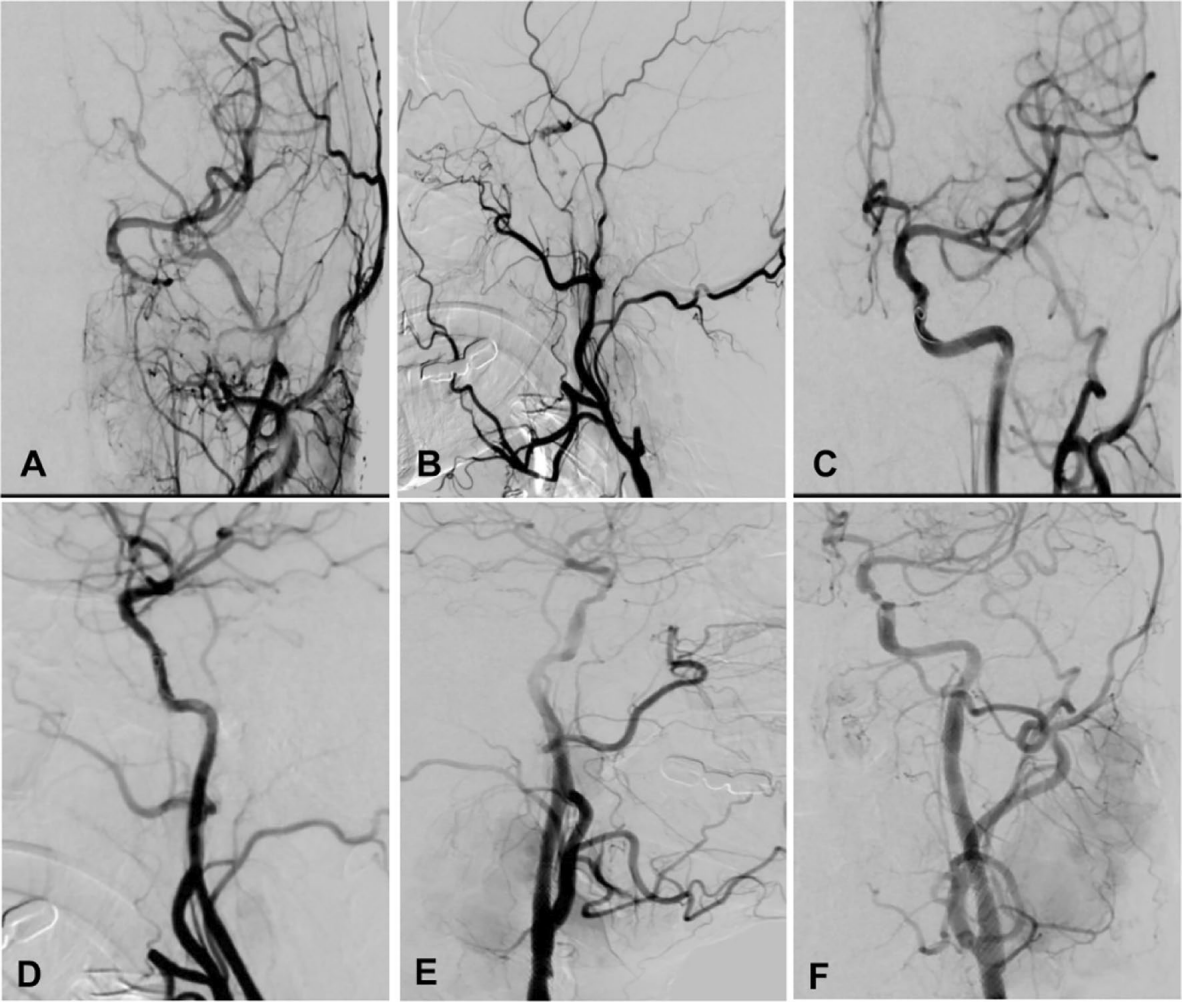
Endovascular recanalization of long-segment atherosclerotic occlusion of the left carotid artery in a patient. (**A** and **B**) Cerebral angiography demonstrated occlusion of the carotid artery on the left. (**C** and **D**) Immediately after endovascular recanalization, the left carotid artery was opened. (**E** and **F**) Follow-up angiography 14 months later showed patent artery.

**Figure 3:**
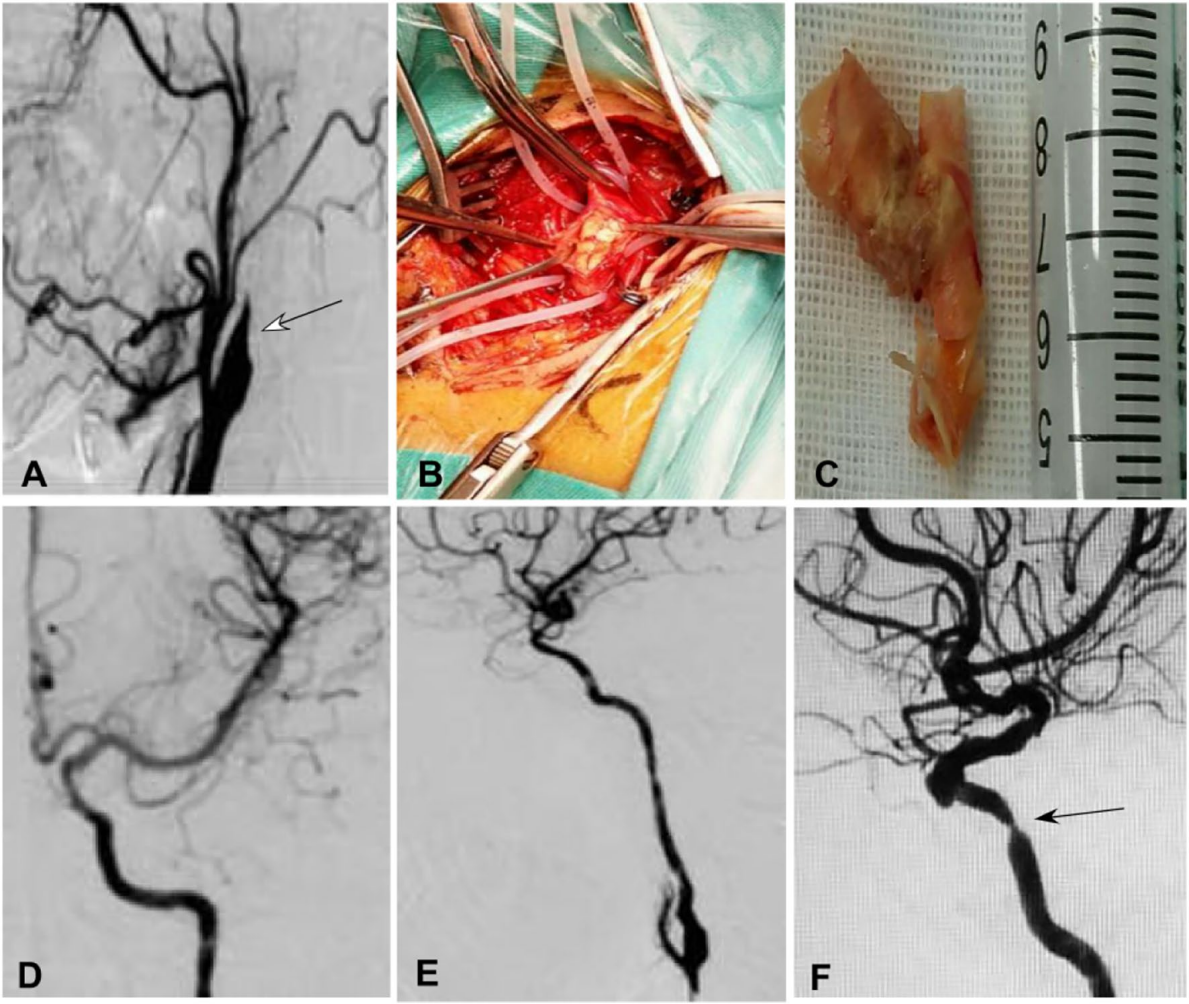
A patient with long-segment carotid artery occlusion. (**A**) Cerebral angiography revealed complete occlusion (arrow) of the left carotid artery occlusion. (**B**) After initial endovascular recanalization failed because a micro-guide wire could not pass through the occluded segment, hybrid surgery was begun with carotid endarterectomy. (**C**) The initial carotid plaque was removed, but distal organized thrombi could not be removed and was treated with stenting. (**D**) After intracranial stents were deployed to compress the distal thrombi onto the arterial wall, cerebral angiography revealed patent carotid artery. (**E**) Immediately after the hybrid surgery, the carotid artery was patent. (**F**) Eight-month follow-up demonstrated a severe stenosis (arrow) at the stented segment which was treated with expansion of a 3.5× 15 mm balloon catheter without successful recanalization.

**Figure 4:**
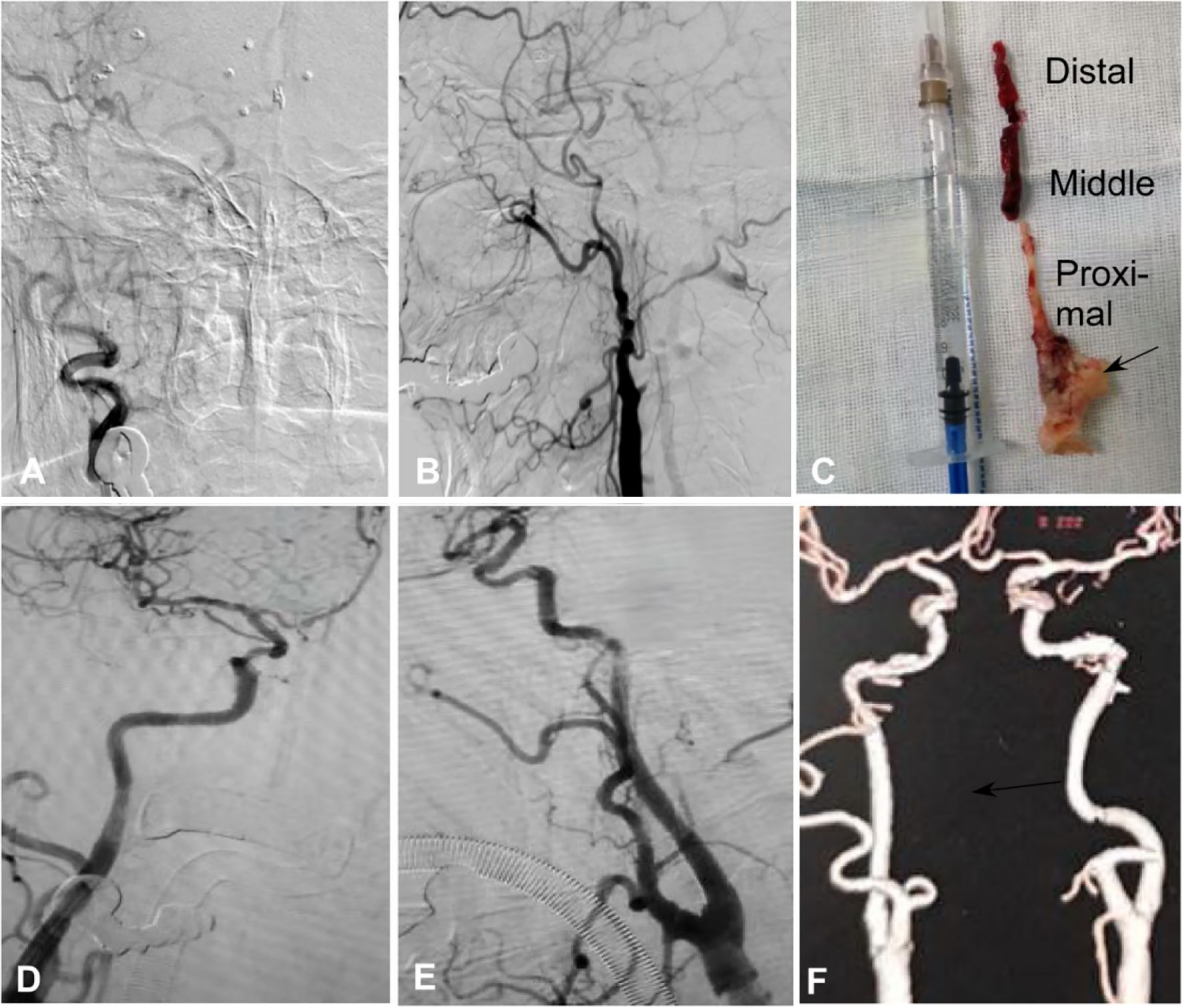
A patient with long-segment occlusion of the right carotid artery. (**A** and **B**) Cerebral angiography showed occlusion of the right carotid artery. (**C**) Carotid endarterectomy was performed to remove the clot occluding the carotid artery. The clot was white organized at the carotid bifurcation and proximal segment of the internal carotid artery (ICA), old dark red in the middle ICA and old red in the distal ICA segment. (**D** and **E**) Immediately after stenting with two stents, the carotid artery was patent. (**F**) Six-month follow-up with computed tomographic angiography demonstrated patient carotid artery.

Hybrid surgery was performed in 44 (50.6%) patients while endovascular recanalization only was conducted in the other 43 (49.4%) (Table 2 and Figs.1, 2, 3, 4). Forty-two (95.5%) patients were successfully recanalized using the hybrid surgery while 40 (93.0%) were successfully opened with the endovascular approach only (Table 3). In patients with type I occlusion, endovascular recanalization only was performed in 22 (47.8%) patients and hybrid surgery in 24 (52.2%), and recanalization was successful in all (100%) patients. In patients with type II ICA occlusion, endovascular recanalization only was conducted in 21 (51.2%) patients and hybrid surgery in 20 (48.8%). Five (12.2%) patients with type II occlusion failed to be recanalized because the micro-guide wire could not be navigated through the occluded segment (Tables 2 and 3). In all patients, the success rate of recanalization was 93.0% (40/43) for the endovascular approach and 95.5% (42/44) for the hybrid surgical approach (Table 4). In patients with type II occlusion, the success rate of recanalization was 85.7% (18/21) for the endovascular approach only but 90% (18/20) for the hybrid surgery. The total success rate of recanalization was 94.3% (82/87) for all patients, 100% for type I occlusion, and 87.8% for type II occlusion. No significant (*P* = 0.12) differences existed in the recanalization rate between groups I and II.

**Table 2:** Treatment approaches for patients.

Variables	Total (87)	Type I occlusion (46)	Type II occlusion (41)	
Endovascular treatment only	43 (49.4%)	22 (47.8%)	21 (51.2%)	0.34
Hybrid surgery	44 (50.6%)	24 (52.2%)	20 (48.8%)	
Recanalized	82 (94.3%)	46 (100%)	36 (87.8%)	0.80
Non-recanalized	5 (5.7%)	0	5 (12.2%)	
Follow-up				
Patients (n)	82 (94.3%)	46 (100%)	36 (87.89%)	NA
Follow-up (m)	6–39 (16)	6–39 (15)	6–37 (16)	0.17
Re-occlusion	4 (4.9%)	0	4 (11.1%)	NA
Good mRS	65 (79.3%)	38 (82.6%)	27 (75%)	0.35
Symptom improvement	75 (91.5%)	43 (93.5%)	32 (88.9%)	0.32
Total Complications	6 (6.9%)	4 (8.7%)	2 (4.9%)	0.39
ICA-cavernous fistula	2 (2.3%)	2 (4.3%)	0	
Recurrent laryngeal nerve injury	2 (2.3%0	2 (4.3%)	0	
Thrombosis	2 (2.3%)	0	2 (4.9%)	

mRS, modified Rankin Scale score; ICA, internal carotid artery.

**Table 3:** Outcomes immediately after surgery and at follow-up.

Variables	Surgical outcomes		Follow-up	
	Recanalized	Non-recanalized	Patent	Re-occluded
Type I occlusion (46)				
Endovascular	22 (47.8%)	0	22 (47.8%)	0
Hybrid surgery	24 (52.2%)	0	24 (52.2%0	0
Total (n)	46 (100%)	0	46 (100%)	0
Type II occlusion (41)				
Endovascular	18 (43.9%)	3 (7.3%)	18 (50%)	0
Hybrid surgery	18 (44.9%)	2 (4.9%)	14 (38.9%)	4 (11.1%)
Total (n)	36 (87.8%)	5 (12.2%)	32 (78.0%)	4 (9.8%)

The total number of patients was 46 with type I occlusion and 41 with type II occlusion. The total number of patients with follow-up was 46 with type I occlusion and 36 with type II occlusion.

**Table 4:** Success rate and complications in endovascular and hybrid approaches.

Variables	Endovascular approach (43)	Hybrid surgery (n = 44)	*P*
Success rate	40/43 (93.0%)	42/44 (95.5%)	0.79
Total complications (n, %)	2 (4.7%)	4 (9.1%)	0.42
Recurrent laryngeal nerve injury (n, %)	0	2 (4.5%)	
ICA-cavernous fistula (n, %)	1 (2.3%)	1 (2.3%)	
Thombosis (n,%)	1 (2.3%)	1 (2.3%)	

ICA, internal carotid artery.

In group I, surgical complications occurred in four (4/46 or 8.7%) patients, including ICA-cavernous fistula in two (4.3%) which was healed at discharge and injury of recurrent laryngeal nerve in two patients (4.3%). In group II, thrombosis occurred in two (4.9%) patients and treated with thrombectomy, resulting in no neurological deficits. A total complication rate was 6.9% in all the patients, with no significant (*P* > 0.05) difference in the complication rate between the two groups. For complications in relation to the treatment approaches, the total complication rate was 4.7% in the endovascular approach and 9.1% in the hybrid approach, and the complication rate was insignificantly (*P* > 0.05) higher in the hybrid than the endovascular approach.

Clinical follow-up was performed in 82 (94.3%) patients 6–39 months (mean 16) after the surgery, with follow-up being performed in all the patients in group I but in 36 (87.8%) in group II. Follow-up was not performed in the five patients who failed the recanalization in group II. The symptoms were improved in 75 (91.5%) patients, and the other seven (8.5%) did not have any improvement in the symptoms. Re-occlusion occurred in 0 in group I but in four (9.8%) in group II (Table 2). Angiographic follow-up was performed with computed tomography angiography in 25 (30.5% or 25/82) patients and digital subtraction angiography in the rest 57 (69.5% or 57/82). Re-occlusion was asymptomatic in two patients and transient ischemic attack in the other two. No significant (*P* = 0.44) differences existed in the re-occlusion rate between groups I and II. The mRS was good (0–2) in 38 (82.6% or 38/46) patients in group I and in 27 (75% or 27/36) patients in group II, with no significant (*P* = 0.78) difference in the mRS between the two groups.

## Discussion

In this study investigating the effect of endovascular and hybrid recanalization on chronic occlusion of the ICA and the effect of occlusion location on the recanalization rate and prognosis, it was found that chronic long-segment ICA occlusion could be safely and efficiently recanalized with the endovascular and hybrid surgery. The location of ICA occlusion may have a critical role in determining the recanalization rate, and careful evaluation of the occlusion location may be helpful in increasing the prognosis of recanalization.

Carotid endarterectomy can be used to directly recanalize narrow or occluded carotid arteries to improve intracranial blood flow supply, but it is only suitable for cases with shorter occluded segments of the ICA, with a success rate of 40.7–87.5% ^20^. Endovascular treatment can be used to open chronic ICA occlusion with longer occlusive segments, with a success rate of 61.6–8.0%^17,21^. For patients with chronic ICA occlusion whose distal end of occlusion was located beyond the ICA cavernous segment, the recanalization rate was low for either endovascular approach alone or carotid endarterectomy alone^20,21^, whereas combination of the endovascular stent angioplasty and carotid endarterectomy may be a feasible approach for successful recanalization. In the study by Jiao et al. including four patients who had long-segment ICA occlusion beyond the cavernous segment, three patients were successfully recanalized using the hybrid surgery^22^. In the study by Zhang et al.^6^ which included 15 patients with long-segment ICA occlusion at or beyond the cavernous segment, the recanalization rate of hybrid surgery was 100%. In our study with long-segment ICA occlusion at or below the clinoid segment, the total success recanalization rate was 94.3%, whereas the success recanalization rate was 95.5% for the hybrid surgery and 93.0% for the endovascular approach. There was no significant (*P* > 0.05) difference in the success recanalization rate between the two treatment approaches. Currently, the use of hybrid surgery for recanalizing long-segment ICA occlusion is still at the initial exploratory stage, with no reports of a large sample of cases.

In our study, the occlusion location of ICA was divided into two types: type I with the occlusion from the neck to the cavernous segment and type II with the location from the neck to the clinoid segment or beyond. Type I occlusion has a shorter, straighter occlusive segment, through which a guide wire can be easily navigated without creating severe complications like arterial dissection. Type II occlusion has a longer tortuous segment, which may create greater surgical difficulties because a guide wire cannot be easily navigated through the tortuous segment after passing through the petrous and cavernous segments. It is also easy to dissect or perforate the ICA wall, leading to intracranial hemorrhagic complications, during maneuver of the guide wire because of the relative thinness of the ICA wall beyond the cavernous segment, especially for patients with severe atherosclerosis. In our study, the total complication rate was 6.9%, including ICA-cavernous fistula in two (4.3%) and injury of recurrent laryngeal nerve in two patients (4.3%) in type I occlusion and thrombosis in two (4.9%) patients in type II occlusion. No neurological deficits or death occurred in the patients with the atherosclerotic ICA occlusion beyond 3 weeks in our study probably because the thrombi within the ICA lumen had become organized beyond this time and were less likely to be shed off and cause symptomatic thromboembolism during therecanalizaiton treatment. The study by Chen et al.^17^ showed that the recanalization rate was 93%, 80%, 73%, 33% and 29% for ICA occlusion below the petrous, cavernous, clinoid, ophthalmic segment, and above the posterior communicating artery, respectively. In our study, the recanalization rate was 100% for type I occlusion below the cavernous segment but 87.8% for type II occlusion from neck to the clinoid segment or beyond even though no significant differences existed in the recanalization rate between the two groups. All five failures of recanalization were of type II occlusion because of the difficulty of the guide wire to pass through the occlusive segment and enter into a false lumen, resulting in recanalization failure. The length of occluded segments, occlusion duration, occlusion shape, and plaque location may also affect the success rate of recanalization^17^.

After endovascular stent angioplasty, the damage of stenting to vascular wall, inflammatory reaction of foreign body (stents) and long-term traction of stent to the artery can lead to intimal proliferation, subsequent thrombosis, restenosis and even re-occlusion of the stented arterial segment. After investigating the endovascular recanalization of 41 patients with chronic ICA occlusion, Lee et al.^23^ reported the rate of technical success, major complications, and re-occlusions within 1-year as 52%, 22%, 91% in patients with ICA occlusion at the clinoid segment (n = 23), and 89%, 0%, 0% in patients with the ICA occlusion proximal to the clinoid segment (n = 19), respectively. In our study, the re-occlusion rate was 0 in type I occlusion but 11.1% in type II occlusion. Re-occlusion may also be related to the arterial diameter, stent size and types to cause intimal proliferatio^24,25^. The study by Xu et al.^24^ showed that the arterial restenosis rate was negatively associated with the arterial dimater, whereas Wasser et al.^25^ found that the narrower and longer a stent deployed in the artery, the higher the incidence of the stent restenosis. In our study, type II occlusion has a longer segment of occlusion and a smaller diameter at the distal occlusive segment, which may require deployment of a longer and narrower stent in the artery, thus resulting in a wider arterial injury and subsequent severe restenosis and more cases of arterial re-occlusion.

Precise presurgical evaluation is the key to successful recanalization of chronic ICA occlusion and post-surgery prognosis^26^. Presurgical assessment of the thrombotic characteristics within the artery, occlusion segment, and collateral formation may be helpful in predicting the rate of successful recanalization, complications and prognosis. Neck vascular ultrasound is helpful for locating the plaque. For a plaque located at the ICA neck segment, an open surgery with (hybrid surgery) or without endovascular management (an open surgery alone) can be used to increase the recanalization rate because an open surgery may be sufficient to remove the plaque without further endovascular operation, even though no evidence has been provided by our study. However, for a plaque located within the ICA intracranial segment, it is difficult to decide whether to use the hybrid or endovascular approach because the recanalization rate of the hybrid surgery was similar to that of endovascular recanalization alone. Nonetheless, the classification of long-segment ICA occlusion is helpful in selection of the surgical plan and evaluation of intraprocedural risk and prognosis. In our opinion, for patients with type I ICA occlusion with plaques located in the intracranial segment, the endovascular recanalization approach can be adopted because it may result in minimal trauma, a low incidence of complications, a low postoperative re-occlusion rate, and good long-term results. For patients with type II occlusion, the hybrid approach can be adopted and may relatively reduce the risk of embolism events although the success rate is comparable to that of endovascular therapy only. Moreover, because the re-occlusion rate of type II ICA occlusion was relatively high, and use of drug-coated stents may be able to reduce the long-term re-occlusion rate. Nonetheless, no studies have been presented in the treatment of ICA occlusion up to the ophthalmic and posterior communicating artery segments using drug-eluted stents.

In our study, chronic atherosclerotic occlusion was defined as ICA occlusion over 3 weeks after imaging confirmation. At this time, most of the thrombi occluding the artery have become stable and organized. Recanalization at a time earlier than 3 weeks may encounter a fresh thrombus and new cerebral infarction, which may result in an increased risk of perioperative thrombus shedding, bleeding transformation, and stent reocclusion based on other studies^3,27,28^. Not all occlusions were detected at the time of occlusion because some patients may have good collateral compensation without causing relevant ischemic symptoms, which may result in the delay of detection and treatment. Treatment in our study was thus performed at a time greater than 3 weeks after imaging confirmation.

Endovascular recanalization has been performed to recanalize chronic total carotid artery occlusion as reported in a small cohort of patients (n = 20) in a recent study^29^, with 30% (n = 6) patients having the occlusion being limited to the cervical segment, 25% (n = 5) to the petrous segment, and 45% (n = 9) to the cavernous segment. This study reported a technical success rate of 80%, a morbidity-mortality rate of 5%, a symptomatic procedure-related complication rate of 30%, and an early stent occlusion of 25% (n = 5), which seemed that the endovascular approach was associated with a much poorer outcome compared to that of ours. Our hospital has a large volume of patients, with thousands of patients of cerebrovascular diseases being enrolled and treated every year due to the large population of China, especially in the local Henan Province. This large volume of patients has made the physicians very proficient in the endovascular and hybrid treatment skills for cerebrovascular disease. Moreover, the patients enrolled in our study were limited to those with the occlusion beyond 3 weeks after imaging confirmation so as to decrease the fresh-thrombus-related procedural complications, morbidity and mortality. Thirdly, pre-surgical evaluation of patients was performed to include different patients with appropriate indications for a suitable treatment approach. These measures may contribute greatly to the greater success rate, greatly decreased complication rates, and much better outcomes in our study from a large hospital with a large volume of patients with cerebrovascular diseases. In comparison of the research outcomes and prognosis, this factor should be taken into consideration because it can make a significant remarkable difference.

Some limitations existed in this study, including the retrospective and one-center study design, no randomization, a small cohort of patients, and Chinese patients enrolled only, which may all affect the outcomes and generalization of this study. Future prospective, randomized, multicenter studies with multiple races enrolled will have to be performed to resolve the above issues for better outcomes.

In conclusion, chronic long-segment ICA occlusion can be safely and efficiently recanalized with the endovascular and hybrid surgery. The location of ICA occlusion may have a critical role in determining the recanalization rate, and careful evaluation of the occlusion location may be helpful in increasing the prognosis of recanalization.

## Data Availability

Data are available from the corresponding author on reasonable request.
